# Enhancements to a Telewellness Program for People With Physical Disabilities: Mindfulness, Exercise, and Nutrition To Optimize Resilience (MENTOR 2.0)

**DOI:** 10.5888/pcd21.230181

**Published:** 2024-01-04

**Authors:** James H. Rimmer, Hui-Ju Young, Vasil Bachiashvili, Navneet Kaur Baidwan, Tapan Mehta

**Affiliations:** 1 School of Health Professions, The University of Alabama at Birmingham, National Center on Health, Physical Activity and Disability; 2 Department of Family and Community Medicine, The University of Alabama at Birmingham

## Abstract

**Introduction:**

This study evaluated the National Center on Health, Physical Activity and Disability (NCHPAD) Mindfulness, Exercise, and Nutrition To Optimize Resilience (MENTOR) program for people with physical disabilities.

**Methods:**

This retrospective evaluation of MENTOR 2.0, an 8-week online group health promotion program, was based on improvements from its first implementation (MENTOR 1.0). Baseline and postassessments included the Godin Leisure-Time Exercise Questionnaire (GLTEQ), NCHPAD Wellness Assessment (NWA), Connor-Davidson Resilience Scale, and Mindfulness Attention Awareness Scale. Estimates and corresponding 95% CIs from linear mixed models were provided to compare baseline and postassessment scores and effect sizes using Cohen *d.*

**Results:**

Among 116 participants (mean age, 53 y; 63% female), postassessment scores increased significantly in the overall NWA and in all 15 NWA domains (effect size, 0.30–0.69). The overall NWA score was 7.59 (95% CI, 5.63–9.56) units higher at postassessment compared with baseline. Scores for GLTEQ health contribution increased significantly among participants with low baseline scores (31.37 [95% CI, 12.97–49.77]) (effect size, 0.50). Mindfulness and resilience scores both showed improvement (0.16 [95% CI, 0.01–0.31]; effect size, 0.15) and (0.72 [95% CI, −0.25 to 1.68]; effect size 0.09), respectively, but only the change in mindfulness was significant.

**Conclusion:**

MENTOR 2.0 advanced the evaluation of this online telewellness program for people with physical disabilities by demonstrating consistent results with MENTOR 1.0. We reported improvements in GLTEQ, especially among those with lower baseline scores; in multiple areas of wellness, including physical, mental, and emotional/spiritual health; and in mindfulness and resilience, although the improvements in these 2 constructs were small.

**Summary**

**What is known on this topic?**

Research shows that the MENTOR (Mindfulness, Exercise, and Nutrition To Optimize Resilience) digital health wellness program is effective in improving wellness for people with physical disabilities who face access barriers to traditional wellness practices.

**What is added by this report?**

We evaluated the MENTOR 2.0 program and reported improvements in multiple areas of wellness including physical, mental, and emotional/spiritual health and noted small improvements in mindfulness and resilience.

**What are the implications for public health practice?**

This quality improvement study adds to the utility and adoptability of the MENTOR program and can be used by program implementers and researchers interested in designing or testing digital health wellness programs for populations with disabilities.

## Introduction

Many people who have a physical disability, defined as a limitation in performing certain activities of daily living or using an assistive device (eg, power or manual wheelchair) (1), often face barriers in engaging in various types of wellness activities (2,3). Community-based health promotion programs (eg, recreation, leisure, proper nutrition, stress management) are often impeded by a community that is underprepared to support their health and wellness needs (4,5). These barriers include inexperienced staff, lack of community support to assist with program and transportation costs, and lack of social support from friends or family (6–11). In addition to these external barriers, many people with disabilities have higher rates of loneliness and social isolation, which could have a negative effect on their participation in leisure time wellness activities (12). In a recent study, Emerson et al (13) reported that, compared with people without disabilities, people with disabilities had a disproportionately higher rate of loneliness (17.2% vs 4.2%) and social isolation (7.1% vs 4.5%), and a lower perceived level of social support (15.5% vs 6.5%).

People with physical disabilities are also more likely to report poor health and experience a higher rate of secondary conditions (eg, pain, fatigue, depression) compared with the general population (14,15). In one scoping review, compared with people without disabilities, people with disabilities reported higher rates of chronic pain (62% vs 14%), fatigue (69% vs 13%), and depression (50% vs 12%) (15). A recent report by the World Health Organization (WHO) noted that people with disabilities have higher rates of premature mortality and morbidity compared with people without disability due to diabetes, heart disease, and mental health conditions and that their access to quality health care is much lower than in the general population (16). The WHO report also noted that people with severe disabilities have a 2.5-fold higher likelihood of depression. Iezzoni et al reported that discriminatory practices in medicine, often referred to as ableism, pervades the US health care system, increasing multiple health disparities and limiting opportunities for people with disabilities to self-manage the conditions that affect their health (17).

Health promotion programs for people with disabilities that are timely, relevant, and accessible are greatly needed to support them and their family members as they transition from health care settings to sustainable health promotion and wellness practices in their homes or communities. These programs can have a substantial impact on reducing health care use, preventing secondary physical and mental conditions, and improving overall quality of life (18,19). Jones et al reported that people with disabilities are underrepresented in mobile health applications and that a strong need exists to develop online health promotion programs that address and mitigate multiple chronic health conditions and risk factors (eg, diabetes, cardiovascular disease, obesity) (20). Both the 2005 Surgeon General’s report on disability and health (21) and the recent 2022 WHO report on disability (16) emphasized the strong need to expand health promotion programs for people with disabilities. The reports recommended greater emphasis on creating or identifying accessible health promotion programs that offer people with disabilities the opportunity to participate in these programs without having to overcome the multiple typical barriers they experience when attempting to access them.

Telehealth, a broad term used to describe the use of telecommunications technology to provide health care services remotely (22), has become a promising approach for reaching people with disabilities who may not have access to community-based health promotion programs. Compared with only a few years ago, millions more people now have internet through cellular networks, and many people choose these over broadband access to the internet (23). Commercial video platforms like Zoom have also become ubiquitous postpandemic, and ownership of smartphones has increased dramatically over the past 5 to 10 years (24).

To offer health promotion programs to more people with disabilities, we developed a specialized telehealth program referred to as MENTOR (Mindfulness, Exercise and Nutrition To Optimize Resilience) that is being used by people with disabilities across the US. MENTOR was established with funding from the Centers for Disease Control and Prevention (CDC) as an online program offered to anyone in the US with internet access and a self-reported mobility disability. This second evaluation of the program, which we refer to as MENTOR 2.0, advances our evaluation of the program based on feedback we received from MENTOR’s first pilot program in 2022 (25).

## Methods

### Design

We used a quasi-experimental design to examine quality improvement in the second implementation phase of the MENTOR program that took place from February 2022 to November 2022. The study was designated and approved by the institutional review board as a quality improvement project (IRB-300008580) of an existing program under the National Center on Health, Physical Activity and Disability (NCHPAD), which did not need trial registration. Data were aggregated across centers using identifiers to prevent loss of confidentiality. The study was conducted in accordance with the Declaration of Helsinki (as revised in 2013), and informed consent was waived due to the retrospective nature of the study (26).

### Description of MENTOR

Participants signed up for an 8-week, 40-hour program (5 h/wk) that delivers online classes on evidence-based wellness practices (ie, health promotion) designed to support participants in improving their health (27). The core wellness domains are mindfulness, exercise, and nutrition. Mindfulness classes are taught once per week for 1 hour by a trained mindfulness instructor and are based on the core elements of mindfulness, including positioning (adapted for wheelchair users), focusing on the breath, and reducing unwanted thought patterns. Exercise classes are offered 2 days per week for 1 hour each class by trained instructors and include movement-to-music (28), complementary alternative medicine (yoga, Pilates, dual-tasking exercises) (29), and general fitness classes. Each class includes adaptations tailored to a participant’s functional level. The nutrition classes are offered once per week for 1 hour by a registered dietitian and focus on key areas of basic nutrition such as preparing meals, increasing fruit and vegetable consumption, shopping and selecting less processed foods, and lowering sugar intake using natural alternatives.

### MENTOR health coaching session

In addition to the 3 core domains, 8 additional wellness domains are briefly discussed with participants during a weekly virtual health coaching session moderated by a trained health coach. Each session is 1 hour. Before the first session, participants receive information on the 11 evidence-based wellness domains of the MENTOR program, using the acronym MY SCORECARD: **M**indfulness, **Y**our spiritual practice, **S**elf-care skills, **C**ore values, **O**utdoor time in nature, **R**elationships, **E**xercise, **C**ontribution to others, **A**rts and leisure, **R**est and relaxation, and **D**iet. During each session, health coaches discuss 1 wellness domain not covered in the core mindfulness, exercise, and nutrition classes. The content includes an overview of the domain’s importance (eg, improving sleep quality, benefits of a spiritual practice, promoting self-care) and how it relates to improving physical, mental, or emotional/spiritual health. MENTOR participants are provided with additional resources before or after the session (via text or video). The objective of each coaching session is to encourage participants to develop their own strategies to engage in a particular wellness behavior to optimize their health.

The MENTOR program uses the latest technology to increase participant engagement. Robust software provides a connected health care model that links people with disabilities to their health coaches, offering them more participation in self-directed care (see Rimmer et al [25] for description of the software used in MENTOR). Social support is particularly important for people with disabilities because they often have higher rates of social isolation and loneliness compared with the general population (13). For this reason, MENTOR was designed as a group-based wellness program that allows participants to engage with one another socially. On average, each class has 15 to 25 participants. After the 8-week program is completed, participants can maintain contact with one another through social media platforms and are encouraged and supported by MENTOR staff.

### Measures

The MENTOR 2.0 program included the same set of quantitative evaluation measures as MENTOR 1.0 (Godin Leisure-Time Exercise Questionnaire [GLTEQ] and wellness assessment) (25). As an enhancement, we included the following additional measures in MENTOR 2.0: a resilience measure (Connor-Davidson Resilience Scale, CD-RISC [30]), which was added to gain a better understanding of whether the program had any benefit to improving resilience, and a mindfulness scale (Mindfulness Attention Awareness Scale, MAAS [31]), which was added to identify potential changes in mindfulness. 

The CD-RISC 10 is a short version of the full CD-RISC 25 and consists of 10 items (32). The response scale has a 5-point range from 0 (not true at all) to 4 (true nearly all the time). Scores are summed to provide a total score of 40, where higher scores indicate higher resilience. The MAAS contains 15 items that ask participants how frequently they have a certain experience, using a 6-point Likert scale from 1 (almost always) to 6 (almost never). The MAAS scale is scored by calculating the mean of these 15 items. Higher scores reflect more mindfulness.

We also modified the comprehensive wellness assessment used in MENTOR 1.0, the NCHPAD Wellness Assessment (NWA), to address the need for clarity of some questions reported by participants and health coaches. Physical, mental, and emotional/spiritual health are the 3 dimensions of overall health that the NWA assesses. Each dimension has 5 questions, and an additional question assesses overall wellness (16 questions total). The NWA is scored on a Likert scale from 1 (very unsatisfied) to 5 (very satisfied). We multiplied each by 10 to provide a calculated score of 10 to 50 per item. The NWA’s face validity was determined through its development by disability fitness experts and people with disabilities during several collaborative iterations, and it serves as a criterion-referenced tool to monitor changes in wellness of the participants at the beginning and end of the program.

Measures of quantitative data also included the GLTEQ, a self-report measure of physical activity. The GLTEQ is a 7-day recall questionnaire that contains 3 questions pertaining to light, moderate, and vigorous intensity activity. The GLTEQ can be scored either by summing all 3 intensity types (Total Activity score) after multiplying each by 3, 5, and 9, respectively, or by summing moderate and vigorous activity only (Health Contribution score). The latter scoring method has been linked with health-enhancing volumes of exercise (33,34). In either method, a score of less than 24 indicates insufficient activity, whereas a score of 24 or higher indicates sufficient physical activity.

### Statistical analysis

Enrollment data regarding reach included the number of people who enrolled, completed, and dropped out of the program; sex; and location (ie, state). Linear mixed models were used to compare baseline and postassessment scores while accounting for the heterogeneity resulting from participants enrolling in different class iterations of the MENTOR program. We provide the Holm step-down, correction-based *P* values, along with *P* values based on linear mixed models. We also provide effect sizes (Cohen *d*) for the outcome measures. Effect size estimates were interpreted as follows: 0.2 = small, 0.5 = medium, and 0.8 = large (35). Participants with missing or incomplete data (eg, did not complete the program or did not complete at least 1 measure preassessment and postassessment) were excluded from the analysis.

MENTOR is open to anyone with a physical disability, including participants who had high starting baseline wellness scores (ie, ceiling effect). Hence, we conducted 2 sets of analyses that included people with high baseline scores and a subset of participants who had low baseline scores. For the GLTEQ, a high baseline score was having a Health Contribution score of 24 or higher, which classified a person as participating in sufficient physical activity to achieve health benefits (34). An NWA response for item 16 (overall wellness) of 4 (satisfied) or 5 (very satisfied), which was converted to a score of 40 to 50, was considered a high baseline score.

## Results

A total of 203 participants were enrolled in MENTOR 2.0 between February and November 2022. Of these, 116 participants completed at least one pre- and post-MENTOR 2.0 assessment (Table 1). The mean age of the participants was 53 years; 63% were female, 61% were White, and 27% were Black. Spinal cord injury was the most common primary disability (16%) and more than one-fifth of participants had multiple disabilities.

**Table 1:** Descriptive Characteristics for Overall Key Study Covariates Among MENTOR 2.0 Participants^a^

Characteristics	N = 116
**Gender**	
Female	73 (62.9)
Male	42 (36.2)
Missing	1 (0.9)
**Age, y**	
Mean (SD)	53.2 (14.1)
Median (min, max)	53.0 (22.0, 92.0)
Interquartile range (Q3–Q1)	22.3 (65.0–42.8)
**Race and ethnicity**	
White or Caucasian	71 (61.2)
Black or African American	31 (26.7)
Multiple	7 (6.0)
Hispanic or Latino	5 (4.3)
Asian or Pacific Islander	1 (0.9)
Other	1 (0.9)
**Primary disability**	
Multiple conditions^b^	26 (22.4)
Spinal cord injury	19 (16.4)
Multiple sclerosis	15 (12.9)
Stroke	12 (10.3)
Traumatic brain injury	7 (6.0)
Cerebral palsy	5 (4.3)
Spina bifida	4 (3.4)
Limb loss or amputation	1 (0.9)
Clubfoot	1 (0.9)
Other	20 (17.2)
Missing	6 (5.2)

a Values are expressed as number (percentage), unless otherwise indicated.

b Participants with 2 or more disabilities.

Although the differences were not significant, the postassessment scores for GLTEQ Total Activity and Health Contribution were higher by 59.47 units (95% CI, −55.01 to 173.95; Holm-adjusted *P* value = .52; effect size, 0.10) and 52.57 units (95% CI, −39.43 to 144.56; Holm-adjusted *P* value = .52; effect size, 0.11), respectively, compared with the baseline scores (Table 2). Among the subgroup of participants with baseline GLTEQ Health Contribution scores less than 24, the Health Contribution scores were significantly greater at postassessment (31.37 [95% CI, 12.97–49.77; Holm-adjusted *P* value = .002]), with an effect size of 0.50 (Table 2).

**Table 2:** Linear Mixed-Effect Model Comparison of GLTEQ Baseline and Postassessment Scores

Variables	Mean difference (95% CI)	*P* value	Holm *P* value^a^	Cohen *d*	Mean difference (95% CI)	*P* value	Holm *P* value^a^	Cohen *d*
	Overall				Baseline health contribution score <24			
Total Activity Score	59.47 (−55.01 to 173.95)	.30	.52	0.10	24.20 (−10.35 to 58.74)	.16	.16	0.20
Health Contribution score	52.57 (−39.43 to 144.56)	.26	.52	0.11	31.37 (12.97 to 49.77)	.001	.002	0.50

Abbreviation: GLTEQ, Godin Leisure-Time Exercise Questionnaire.

a Holm step-down, correction-based *P* value.

The individual NWA scores at postassessment were significantly greater than the baseline scores (Table 3). Among the physical health domain scores, the greatest differences between the baseline and postassessment scores were observed for exercise (9.13 [95% CI, 6.81–11.44; *P* < .001]) and nutrition (7.09 [95% CI, 5.05–9.14; *P* < .001]). Among the mental health domain scores, the postassessment scores were 6.12 (95% CI, 4.01–8.24; *P* < .001) units higher in core values and 5.31 (95% CI, 3.13–7.49; *P* < .001) units higher in self-care. For the emotional/spiritual health domain, contribution to society or community (6.30 [95% CI, 4.05–8.56; *P* < .001]) and outdoor time in nature (6.32 [95% CI, 4.22–8.42; *P* < .001]) showed greatest improvement postassessment. The effect sizes for the NWA items ranged from 0.30 to 0.69. The NWA overall summary score was 7.59 (95% CI, 5.63–9.56; *P* < .001) units higher at postassessment when compared with baseline assessment, and the effect size was 0.66. Among the subgroup with overall NWA scores less than 30, similar trends were observed. Exercise and nutrition scores showed most improvement postassessment, followed by relationships, outdoor time in nature, sleep, and core values. The corresponding effect size for the overall NWA score was 1.84 and the item-specific effect sizes ranged from 0.38 to 0.89 (Table 3).

**Table 3:** Linear Mixed-Effect Model Comparison of NWA Baseline and Postassessment Scores

Variable	Mean difference (95% CI)	*P* value	Holm *P* value^a^	Cohen *d*	Mean difference (95% CI)	*P* value	Holm *P* value^a^	Cohen *d*
	Overall				Baseline overall NWA score <30			
**Physical health**								
Exercise (Q1)	9.13 (6.81–11.44)	<.001	<.001	0.69	10.72 (6.71–14.73)	<.001	<.001	0.87
Nutrition (Q2)	7.09 (5.05–9.14)	<.001	<.001	0.59	10.92 (7.35–14.49)	<.001	<.001	0.89
Self-care (Q3)	3.89 (1.66–6.13)	<.001	.001	0.3	7.5 (3.43–11.57)	<.001	.005	0.53
Sleep (Q4)	4.95 (2.74–7.15)	<.001	<.001	0.37	8.41 (4.38–12.44)	<.001	.001	0.61
Pain management (Q5)	5.69 (3.54–7.83)	<.001	<.001	0.44	6.14 (1.94–10.34)	.005	.01	0.45
**Mental health**								
Managing negative thoughts (Q6)	4.17 (1.92–6.43)	<.001	.001	0.31	6.35 (2.14–10.56)	.004	.01	0.48
Core values (Q7)	6.12 (4.01–8.24)	<.001	<.001	0.51	8.41 (4.41–12.41)	<.001	.001	0.67
Self-care (Q8)	5.31 (3.13–7.49)	<.001	<.001	0.42	5.57 (1.12–10.01)	.02	.02	0.38
Depression and loneliness (Q9)	4.54 (2.46–6.62)	<.001	<.001	0.36	6.71 (2.87–10.55)	.001	.006	0.54
Hobbies (Q10)	4.34 (1.90–6.78)	<.001	.001	0.31	7.92 (3.59–12.25)	<.001	.005	0.53
**Emotional health**								
Inner peace (Q11)	4.69 (2.48–6.91)	<.001	<.001	0.37	7.79 (3.46–12.13)	<.001	.006	0.58
Contribution to society or community (Q12)	6.3 (4.05–8.56)	<.001	<.001	0.47	7.04 (2.86–11.23)	.002	.008	0.53
Spiritual practice (Q13)	3.94 (1.95–5.92)	<.001	<.001	0.33	6.85 (2.74–10.97)	.002	.008	0.5
Relationships (Q14)	4.73 (2.47–6.98)	<.001	<.001	0.38	9.8 (5.97–13.64)	<.001	<.001	0.81
Outdoor time in nature (Q15)	6.32 (4.22–8.42)	<.001	<.001	0.48	8.03 (4.24–11.81)	<.001	.001	0.65
Overall score (Q16)	7.59 (5.63–9.56)	<.001	<.001	0.66	15.51 (12.79–18.24)	<.001	<.001	1.84

Abbreviations: NWA, National Center on Health Physical Activity and Disability Wellness Assessment; Q, question number.

a Holm step-down, correction-based *P* value.

The postprogram MAAS score was 0.16 units higher than at baseline (95% CI, 0.01–0.31), with an effect size of 0.15. The CD-RISC score at postassessment was also higher, but the difference was not significant (0.72 [95% CI, −0.25 to 1.68]; effect size, 0.09).

## Discussion

The findings from MENTOR 2.0 included results from several additions to MENTOR 1.0. The 2 new measures, CD-RISC 10 and MAAS, were added to assess mindfulness and resilience because we considered a primary end point of MENTOR to teach participants how to overcome adversity and adapt to their current health status and environment. Although we did not observe significant changes in CD-RISC 10 and MAAS scores postprogram, assessing its impact on mindfulness and resilience provides useful information for us to continue enhancing the delivery of MENTOR. It also creates new avenues for additional evaluations in the field of wellness and disability. 

Compared with MENTOR 1.0, we observed larger and more consistent improvements in the global wellness assessment (ie, NWA) across all domains, and these improvements were significant. As observed in MENTOR 1.0, no significant increase was seen in physical activity for participants with average to high baseline GLTEQ scores. However, changes in the Total Activity and Health Contribution scores were significant for participants with low baseline GLTEQ scores.

Another goal of MENTOR 2.0 was to obtain a higher completion rate in postprogram assessments. When we compared our current results to the MENTOR 1.0 evaluation, we observed that the percentage of participants completing the postprogram assessments improved from 35% to 56%. We used a few methods to achieve this. First, after reports from MENTOR 1.0 participants that questionnaires were too long, we were able to remove 29 items from the longest questionnaire (nutrition, with 74 questions originally), or a 38% reduction, without compromising the validity of the instrument. Second, the health coaches reminded participants toward the end of the program to complete the postassessments, noting the information would be valuable to them in terms of identifying where they made improvements and what they might want to continue working on post-MENTOR. Third, our rehabilitation center partners who were funded through a subcontract to enroll a defined number of participants were informed about the requirement for each participant to complete both baseline and postassessments. Finally, the health coaches sent out reminders through the Healthie portal (Healthie, Inc) the last week of class to complete the postassessments.

The results of MENTOR 2.0 demonstrated significantly higher improvements in all areas of physical, mental, and emotional/spiritual health in participants who had average to above average wellness scores at baseline. In MENTOR 1.0, we observed significant changes only in 2 wellness domains: exercise and contribution to others. We believe that the improved results may be due to the health coaches becoming more knowledgeable about the program and focusing more on specific wellness domains that had greater alignment with participants’ needs.

One area of interest was examining data on participants who had low baseline wellness scores. Although we saw consistent improvements in this subgroup between MENTOR 1.0 and 2.0 participants, MENTOR 2.0 participants had slightly better improvements. In MENTOR 1.0, only 1 of the 16 wellness-related questions across all participants demonstrated significant gains, and 8 of the 16 wellness-related questions in participants with low NWA baseline scores demonstrated significant gains. Conversely, in MENTOR 2.0, all 16 wellness questions demonstrated significant gains.

### Limitations

Similar to MENTOR 1.0, we saw a potential ceiling effect as we offered the program to any participant with a disability, including those who already had high wellness scores, increasing the likelihood of a ceiling effect for several participants. The study is open to anyone who identifies as having a mobility disability, so whether these findings would generalize to a specific disability group (eg, multiple sclerosis, Parkinson disease) is unclear. Another limitation of the study was the use of a nonrandomized, single-group design, which limited our ability to definitively attribute observed changes solely to the MENTOR program itself. However, the positive findings warrant further investigation, and we recommend incorporating a control group into future research using this program. Findings are also not generalizable to people with intellectual disabilities since the program has not been adapted for this population, although plans are under way to begin adapting a version of MENTOR for them. Future research on this program using instruments that do not have a ceiling effect may provide better results for people who begin the program with higher baseline wellness.

### Conclusion and future directions

Over the past 20 years, the evidence has continued to grow on the importance and effectiveness of noninvasive interventions (ie, exercise, nutrition, mindfulness) in the promotion of lifelong health and wellness (36). As scientists expand their understanding on the importance of integrative wellness and the effect that multiple physical and psychological wellness domains can have on protecting a person’s health, MENTOR will become more precise and individualized for people with varying types of disabilities.

We plan to follow up with former MENTOR participants at 1 year to reassess their health behaviors and determine if the MENTOR program has had any long-term effects on their health and where they possibly need more assistance. When participants sign up for the MENTOR program, they also agree to be part of a longitudinal database so that we can query their health behaviors over the next 3 to 5 years. We have also added an online MENTOR Coffee Club option for post-MENTOR participants who would like to continue meeting in a group to discuss popular topics in wellness (eg, diet, mindfulness, art).

A MENTOR app will be developed that will allow participants to track individual health behaviors in real time over a 24-hour period and provide MENTOR health coaches access to the data. Acquiring and maintaining certain health behaviors is a dynamic process, which is why it is necessary for participants to understand changes in their health as they relate to changes in their behavior or environment. Therefore, getting a snapshot of a person’s wellness at the beginning and end of a program may not be representative of their day-to-day health behaviors. Access to a participant’s wellness practices in real time will allow researchers and practitioners to address issues when they occur, to better identify gaps and inefficiencies that lead to or worsen certain health conditions.
